# Efficacy of Haloxyfop‐R‐Methyl on *Allium cepa*: Cyto‐Genotoxic and *In Silico* Docking Studies on the Mechanism of Action

**DOI:** 10.1002/jemt.24824

**Published:** 2025-03-06

**Authors:** Recep Liman, Emine Kılıç, Erman Salih İstifli, Yudum Yeltekin Uğur, İbrahim Hakkı Ciğerci

**Affiliations:** ^1^ Molecular Biology and Genetics Department, Faculty of Engineering and Natural Sciences Uşak University Uşak Turkiye; ^2^ Biology Department, Faculty of Science and Literature Cukurova University Adana Turkiye; ^3^ Molecular Biology and Genetics Department, Faculty of Science and Literatures Afyon Kocatepe University Afyon Turkiye

**Keywords:** chromosomal aberrations, DNA damage, haloxyfop, mitotic index, molecular docking

## Abstract

Haloxyfop‐R‐methyl (HRM) is extensively employed to control annual and perennial grass weeds. This study aimed to investigate the cyto‐genotoxic effects of HRM at concentrations of 1.19, 2.38, and 4.76 mg/L over various time intervals (24, 48, 72, and 96 h) on *Allium cepa* root. Parameters including root growth, mitotic index (MI), chromosomal aberrations (CAs), and DNA damage were assessed using root growth inhibition, *A. cepa* ana–telophase, and alkaline comet assays. Moreover, to gain molecular insights into the cyto‐genotoxic effects of HRM as well as the active agent haloxyfop‐R (HR), molecular docking was performed against two intracellular target receptors: the carboxyltransferase (CT) domain of yeast acetyl‐CoA carboxylase (ACC) and a double‐stranded DNA dodecamer. The root growth inhibition test revealed a statistically significant reduction in onion root length, from 3.64 ± 0.18 cm at lower concentrations to 0.05 ± 0.02 cm at higher concentrations of HRM. A significant decrease in the MI was observed at all treatment periods, except at 1.19 mg/L after 72 h, along with an increase in CAs during the 24 and 48 h applications, except at 4.76 mg/L after 48 h, in *A. cepa* root cells treated with HRM, compared to the negative control group. DNA damage increased with HRM exposure and was found to be both concentration‐and time‐dependent. Docking studies revealed strong binding interactions of HRM and HR with the CT domain of the ACC enzyme, which is a central player in fatty acid biosynthesis, and showed that both HRM and HR bound specifically to DNA minor groove regions rich in GC bases. The current study confirmed the cyto‐genotoxic effects of HRM. Its use should be carefully regulated, as it may present ecological risks by negatively impacting the genomes of non‐target organisms.

## Graphical abstract

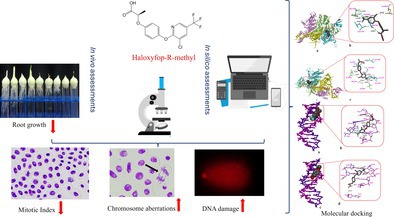

**Summary**

- Haloxyfop‐R‐methyl significantly inhibited onion root growth.
- Allium and comet assays were used.
- Haloxyfop‐R‐methyl has cyto‐genotoxic effects on *A. cepa* root tip cells.
- Molecular docking simulations reveal the interactions between haloxyfop‐R‐methyl and target molecules.

## 1 Introduction

Herbicides are a type of pesticide used to control weeds, with over 400 different chemical structures utilized globally. Aryloxyphenoxy‐propionate (AOPP) group herbicides, which are based on 4‐oxyphenoxypropanoic acid as their core structure, rank among the most widely used herbicide classes globally, second only to glyphosate (Zhou et al. 2018). Currently, the AOPPs dominating the market include cyhalofop‐butyl, diclofop‐methyl, fenoxaprop‐P‐ethyl, quizalofop‐P‐ethyl, fluazifop‐P‐butyl, and haloxyfop‐R‐methyl (HRM) (Wang et al. 2017). They are extensively utilized for controlling annual and perennial grasses in crops such as potatoes, sugar beets, vegetables, and other broad‐leafed plants. These herbicides are absorbed through roots and the leaf surface and distributed throughout the plant, where they inhibit acetyl‐CoA carboxylases (ACCs) (Adkins 1999). HRM, a low‐toxicity aryloxyphenoxypropionate herbicide, is noted for its high efficiency, low toxicity, crop safety, and long‐lasting effects. It is extensively employed to control annual and perennial grass weeds such as *Sorghum halepense* , *Cynodon dactylon* , *Avena fatua* , *Agropyron* sp., *Echinochloa crus‐galli* , *Lolium* sp., *Poa annua* , *Alopecurus myosuroides*, and *Phalaris paradoxa* in crops such as cotton, citrus, lentil, tomato, pepper, soybean, watermelon, sugar beet, onion, rape, peanut, and so forth (Koksal et al. 2018; Wang et al. 2017) HRM works by disrupting lipogenesis, as it prevents the ATP‐dependent carboxylation of acetyl‐CoA from being catalyzed to malonyl‐CoA and inducing oxidative stress by the generation of reactive oxygen species (ROS) in target plants (Banas et al. 2011; Burton et al. 1991; Ore and Olayinka 2019; Zhang et al. 2004).

The Allium test is widely used for evaluating the cyto‐genotoxic effects of herbicides due to its ease of handling, cost‐effectiveness, high sensitivity, favorable chromosomal condition (large and few numbers of chromosomes), and ability to yield meaningful results. It also shows good correlation with other test systems. This test enables the evaluation of various biomarkers, including root growth and mitotic index (MI) for assessing cytotoxicity, chromosomal structural and numerical changes for genotoxicity detection, and the frequency of micronuclei to verify mutagenicity and genotoxicity detection (Amac and Liman 2021; da Silva Souza et al. 2024; Felisbino et al. 2018; Rosculete et al. 2018; Santos et al. 2023; Yüzbaşıoğlu et al. 2009). DNA strand breakage in single cells can be determined using the well‐researched, straightforward, affordable, and sensitive single‐cell gel electrophoresis (SCGE) or the comet assay technique. *A. cepa* root meristem cells are widely used in the comet assay in addition to the Allium test because of their ease of use, quickness, cost, and independence from mitosis for evaluating the genotoxic effects of herbicides (Aydin and Liman 2020; Karaismailoglu 2015; Liman et al. 2015; Ozel et al. 2022; Silveira et al. 2017).

In recent years, the *in silico* molecular docking technique has been widely employed for the elucidation of the molecular mechanisms underlying the cytotoxic and genotoxic effects of synthetic small molecules (Kumari et al. 2017; Ranjan et al. 2018; Ricci and Netz 2009; Saputra et al. 2021). Widely applied in the biological sciences, this computational method provides valuable quantitative insights into the optimal binding modes and binding energetics of small molecules (i.e., pesticides) with intracellular targets, typically proteins/enzymes or nucleic acids. Most importantly, it enables the molecular‐level characterization of the ultimate biological response arising from chemical interventions (Jeong et al. 2019; Liu et al. 2018). In this context, elucidating the intracellular macromolecular targets and intermolecular interactions of the HRM herbicide is crucial for predicting the ultimate biological response in target or non‐target organisms exposed to this herbicide.

Since herbicides are the most used pesticides in crop cultivation, it is crucial to study and assess their environmental impact while exploring alternatives to reduce their large‐scale use. Thus, the aim of this study was to assess the cyto‐genotoxic and DNA‐damaging potential of the herbicide HRM on *A. cepa* root meristematic cells using root growth inhibition, Allium ana–telophase, and comet assays. Additionally, potential receptor–ligand interactions of HRM and its active form in the plant organism, haloxyfop‐R (HR), were elucidated for the first time using the molecular docking technique, providing a mechanistic perspective on the cytotoxic and genotoxic effects caused by this herbicide. In the docking experiments, the carboxyltransferase (CT) domain of yeast ACC and a synthetic double‐stranded DNA (dsDNA) dodecamer (B‐DNA) structure served as the target receptors.

## 2 Materials and Methods

### 2.1 Chemicals

The herbicide Gayder 108 EC containing 108 g/L HRM was purchased from Astronova (Turkey). Table 1 provides some properties of HRM. Additional analytical‐grade chemicals were purchased from Sigma Aldrich (Germany), including Triton X‐100, ethidium bromide, methyl methanesulfonate (MMS), glacial acetic acid, potassium disulfide, trizma hydrochloride, normal and melting agarose, magnesium chloride, EDTA, and sodium and potassium chloride.

**TABLE 1:** Some properties of HRM.

IUPAC name	Methyl (2*R*)‐2‐[4‐[3‐chloro‐5‐(trifluoromethyl)pyridin‐2‐yl]oxyphenoxy]propanoate
Synonyms	Haloxyfop‐P‐methyl, (R)‐methyl 2‐(4‐((3‐chloro‐5‐(trifluoromethyl)pyridin‐2‐yl)oxy)phenoxy)propanoate, Gallant Super, (R)‐haloxyfop methyl ester
Chemical structure	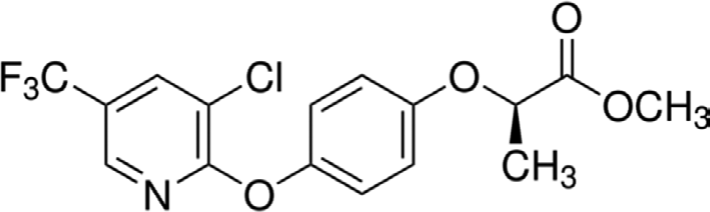
Molecular formula	C1_6_H_13_ClF_3_NO_4_
Molecular weight	375.72 g/mol
CAS number	72619‐32‐0
Water solubility	8.74 mg/L at 20°C

### 2.2 *A. cepa* Root Growth Inhibition Test and EC_50_ Determination

Healthy and equal‐sized (diameter 2–3 cm) *A. cepa* bulbs (2*n* = 16 chromosomes) were purchased from the local market. The root growth inhibition and effective concentration resulting in 50% inhibition of root length (EC_50_) value of HRM for *A. cepa* were carried out using the guidelines provided by Fiskesjö (1985) with adjustments recommended by Kucuk and Liman (2018). HRM was diluted in distilled water at concentrations of 0.5, 1, 2, 2.5, 5, 10, 25, and 50 mg/L, and distilled water was used as the control treatment. The bulbs that had been cleared of their brown outer shells and dried roots were then subjected to several doses of HRM for 96 h while being kept at room temperature in the dark. After 4 days of exposure, the average length of 10 roots from each onion (in total 50 roots for each concentration) was measured using a centimeter scale to assess the harmful effects of HRM. By comparing the average root lengths of the test and control groups, the EC_50_ value of HRM for *A. cepa* was determined as 2.38 mg/L.

### 2.3 Allium Ana–Telophase Test

The Allium ana–telophase test was carried out with slight modifications as outlined by Rank and Nielsen (1994) and according to Aydin and Liman (2020). Onions were cultivated in distilled water in the dark at room temperature for 48 h to develop roots measuring 1–2 cm in length. The *A. cepa* roots were subsequently exposed to HRM at concentrations of 2 × EC_50_, EC_50_, and ½ × EC_50_, along with a positive control ((methyl methanesulfonate‐MMS, 10 mg/L) and a negative control (distilled water), for 24, 48, 72, and 96 h in the dark, at room temperature. The roots were excised and subsequently fixed in Carnoy's fixative solution, composed of ethanol and glacial acetic acid in a ratio of 3:1 (v/v), at 4°C for a duration of 24 h. On the following day, the fixative was changed to 70% ethanol and stored at 4°C until used. The fixed roots were rinsed with distilled water and then hydrolyzed at least 8–10 min in 1 N HCl prewarmed to 60°C. The squash method described by Liman et al. (2021) was followed, staining the root tips with Schiff's reagent for 25 min. Five root tips were used per treatment dose and control group. The following equations were used to analyze about 1000–1060 cells for MI and mitotic phases and 100 ana‐telophase cells (if feasible) for chromosomal aberrations (CAs) for each treatment by using a Nikon Eclipse Ci‐L light microscope (Japan) connected to a CMOS camera (Argenit, Kameram5, Turkey).

$$\mathrm{MI}\;\left(\%\right)=\frac{\text{Total number of dividing cells}}{\text{Total number of cells}}\times 100$$

$$P\text{hase index}\;\left(\%\right)=\frac{\text{Particular phase}\;}{\text{Total number of dividing cells}}\times 100$$

$$\mathrm{CA}\;\left(\%\right)=\frac{\text{Total aberrant cells}}{100\;\mathrm{ana}-\text{telophase cells}}\times 100$$

### 2.4 Alkaline Comet Assay

**FIGURE 1:**
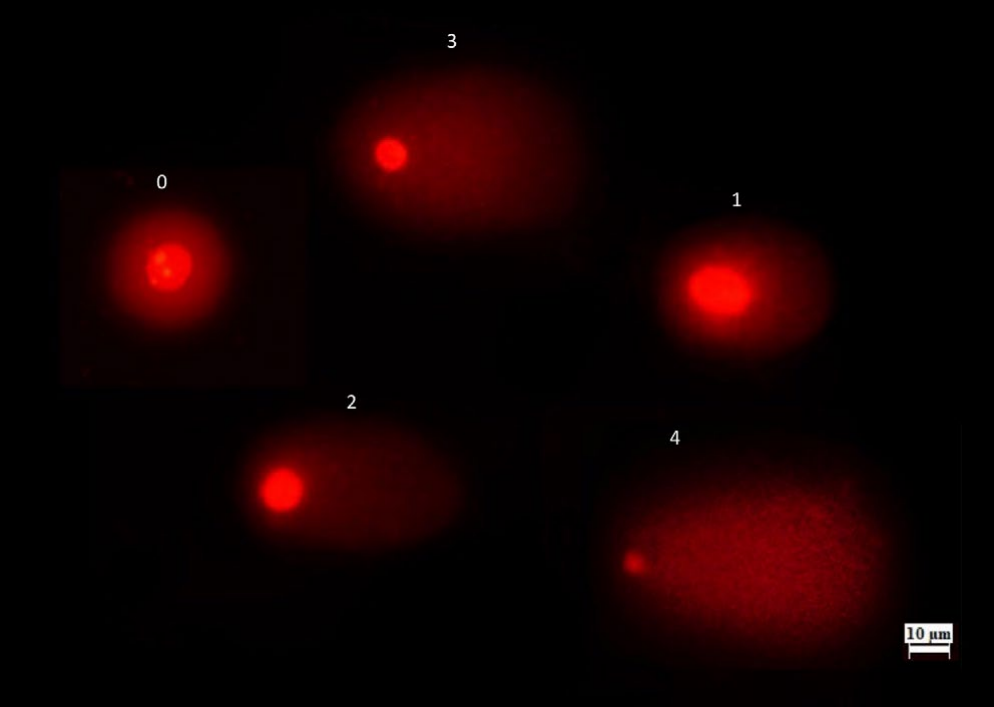
Comet scores of DNA damage induced by HRM in *Allium cepa* roots. 0—no damage (< 5%), 1— minor damage (5%–20%), 2—moderate damage (20%–40%), 3—severe damage (40%–85%), and 4—complete DNA damage (> 85%).

DNA damage in onion root cells induced HRM was detected using a modified comet assay, as described by Tice et al. (2000). Onions were cultivated in distilled water for 2–3 days in the dark at room temperature to promote root growth. They were then exposed to HRM at concentrations of 1.19 mg/L, 2.38 mg/L, and 4.76 mg/L for 24, 48, 72 and 96 hours. Distilled water served as the negative control, while 10 mg/L MMS was used as positive control. 600 μL of ice‐cold nuclei isolation buffer (4 mM MgCl_2_ × 6H_2_O, 200 mM Tris, 0.5% Triton‐X) was added after the tips were finely chopped into 1 cm pieces with a scalpel. After filtration of the onion root cells, the cells were then centrifuged for 7 min at 1200 rpm. Slides were prepared as previously described in Yardimci et al. (2022). A 1:1 (v/v) mixture of 100 μL of nuclear suspension and 1.5% LMPA was spread on a 1% normal point agarose‐coated glass slide and immediately covered with a coverslip. Slides were stored for 20 min at 4°C in an alkaline buffer (1 mM EDTA, 300 mM NaOH, pH > 13) and then subjected to electrophoresis at 25 V and 300 mA for 20 min at 4°C. Lastly, three slides were stained with ethidium bromide (20 μg/mL), and 150 cells per sample (50 cells per slide) were scored using fluorescence microscopy (TAM‐F, Turkey). For each slide, levels of DNA damage were analyzed by visual scoring of comets (0–200 arbitrary unit scale) into five different classes, as shown in Figure 1. A score of 0 represents no damage (< 5%), 1 indicates minor damage (5%–20%), 2 signifies moderate damage (20%–40%), 3 denotes severe damage (40%–85%), and 4 indicates complete DNA damage (> 85%). The arbitrary unit was calculated using the following equation:

$$\text{Arbitrary unit}=\sum_{i=0}^{4}\mathrm{Ni}\times i$$

where *Ni* is the number of cells and *i* is the degree of DNA damage as measured by a score (0, 1, 2, 3, 4).

### 2.5 Molecular Docking Studies

**FIGURE 2:**
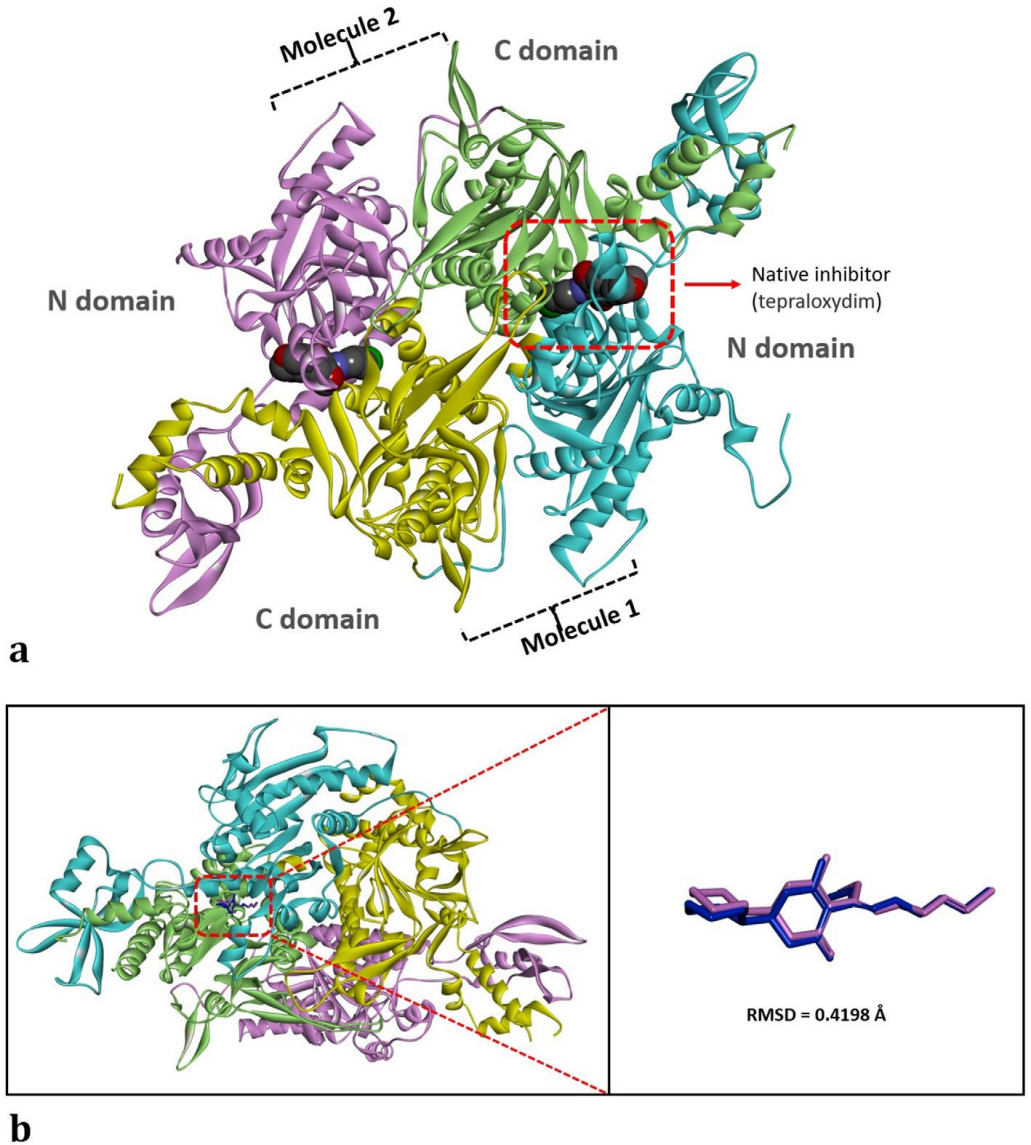
(a) Schematic representation of the CT domain of the yeast ACC enzyme in complex with tepraloxydim. The N domains of both monomers are depicted in cyan and magenta, and the C domains are colored green and yellow. Experimental inhibitor (tepraloxydim) is shown in CPK mode, and the dimeric CT domain is shown in solid ribbon mode. (b) Validation of the docking protocol. The crystal inhibitor tepraloxydim bound to the CT domain of the yeast ACC enzyme in the x‐ray crystal structure was extracted and redocked. The resulting redocking pose (blue ligand) was superimposed onto the original bound structure (pink ligand), and the root mean square deviation (RMSD) was calculated (0.4198 Å). In the figure, the CT domain is presented in solid ribbon mode (left panel), while the bound inhibitor tepraloxydim is depicted in stick mode (right panel). The images were prepared using the BIOVIA Discovery Studio Visualizer v16 program.

This work conducted molecular docking studies to investigate the molecular‐level cyto‐genotoxic effects caused by HRM in *A. cepa* root tip cells. In both plants and soil, HRM rapidly undergoes hydrolysis to form HR, the principal stereoisomer responsible for herbicidal activity (Buerge et al. 2015; Lehner et al. 2020; Poiger et al. 2015). Therefore, following the treatment of plants with HRM, the simultaneous systemic circulation of HRM (pro‐herbicide) and HR (active herbicide) in the plant organism could be expected. Consequently, docking studies were conducted for both HRM and HR. To elucidate the molecular mechanisms underlying the cytotoxic effects of HRM and HR, we focused on haloxyfop's inhibition of the CT domain of yeast ACC (Figure 2a). The function of this domain is essential in the metabolic process of fatty acids, as it facilitates the conversion of acetyl‐CoA into malonyl‐CoA in the cytosol of herb cells (Zhang et al. 2004). Therefore, the potent inhibition of the ACC enzyme's CT domain by HRM and HR can significantly reduce cellular fatty acid levels, thereby disrupting vital cellular functions such as membrane fluidity and cell proliferation, potentially leading to cytotoxicity (Beckers et al. 2007). For the docking experiments, the crystal structure of the yeast ACC enzyme's CT domain (PDB ID: 3K8X, resolution: 2.30 Å) was employed as the primary target receptor. The two different conformations of haloxyfop (HRM and HR) were manually drawn in 2D using ChemSketch (version 2023 1.1), then optimized and converted into 3D structures (Mulatsari et al. 2021). Additionally, the positive control MMS, used in the *A. cepa* test, was downloaded in sdf format from the PubChem database (Compound CID: 4156). To further comprehend the molecular mechanism underlying the observed DNA fragmentation in the comet assay and to calculate binding affinities and gain insights into the resulting intermolecular interactions with DNA, we also conducted molecular docking studies of HRM and HR against DNA. A high‐resolution X‐ray crystal structure of a double‐stranded B‐DNA dodecamer (PDB ID: 8CE2, resolution: 1.24 Å) obtained from the Nucleic Acid Knowledgebase (NAKB) was used for this purpose (Tito et al. 2023).

#### 2.5.1 Preparation of Protein, Nucleic Acid (DNA), and Ligand Structures

Before starting the docking simulations, the Discovery Studio Visualizer program was used to remove water molecules, inhibitors, and non‐interacting ions from the crystal structures of the receptor molecules (CT domain and dsDNA dodecamer) (Biovia 2016). Furthermore, the “Dock Prep” module of the UCSF Chimera program (version 1.17.1) was utilized to add missing atoms to the amino acid side chains and assign molecular charges to the CT domain, and the prepared molecule was then saved in mol2 format (Pettersen et al. 2004). Subsequently, the CT domain was ionized at pH 7.4 using the PROPKA module in the Vega ZZ program (version 3.2.2.21), followed by energy minimization in the NAMD module of the program employing the CHARMM force field (number of time steps: 10.000; temperature: 300 K) (Pedretti et al. 2004). The optimized final 3D structure, representing the “lowest energy conformer” was then saved in pdb format prior to docking. Energy minimization of the ligands HRM, HR, and MMS (experimental positive control) was conducted using the Avogadro program with the MMFF94 force field via the steepest descent algorithm (convergence = 10^−7^). Finally, the structures of the ligands (HRM, HR, and MMS) and receptors (CT domain and DNA) were converted to pdbqt format in the AutoDock Tools 1.5.6 interface before docking experiments (Morris et al. 2009).

#### 2.5.2 Validation of the Molecular Docking Protocol

The ability of the search algorithm in docking studies to accurately generate the binding pose of the experimental ligand (reference ligand) is important for the reliability of the obtained docking poses (Rossino et al. 2020). Therefore, the crystallographic inhibitor (tepraloxydim) bound to the CT domain of the ACC enzyme (PDB ID: 3K8X) (Figure 2b) was extracted from the CT domain and subsequently redocked into the active site (the interface of the dimer) of the CT domain (Figure 2b). Subsequently, the root mean square deviation (RMSD) value of the obtained redocking pose was calculated by superimposing it onto the crystallographic pose, revealing a value of 0.4198 Å (< 2 Å) (Figure 2b). Therefore, our redocking experiment demonstrated that the used docking conformational sampling algorithm could accurately reproduce the binding mode of the crystallographic inhibitor, tepraloxydim (Figure 2b).

#### 2.5.3 Molecular Docking

In this study, molecular docking was conducted using AutoDock Vina (version 1.2.5) to explore the interactions of HRM, HR, and MMS with the CT domain of the yeast ACC enzyme and a dsDNA dodecamer (Eberhardt et al. 2021). AutoDock Tools 1.5.6 was utilized to merge non‐polar hydrogens, while polar hydrogen atoms in the receptors and ligands (HRM, HR, and MMS) were kept during the docking preparations. Kollman charges were assigned to the receptor molecules, whereas Gasteiger charges were allocated to the ligands. The docking simulations were conducted with the rotatable bonds of all ligands set to rotate freely (flexible docking), while the receptor molecules were kept rigid (semi‐flexible docking). For the CT domain, the grid box was configured with dimensions of 18 × 18 × 18 Å (*x*: 46.93; *y*: −35.49; *z*: 19.10), and for the DNA receptor, the grid box size was set to 80 × 70 × 100 Å (*x*: 15.07; *y*: 21.00; *z*: 8.58). In order to ensure that the ligands (HRM, HR, and MMS) could interact with the catalytic residues in the ACC enzyme's CT domain as well as the entire molecular surface of the DNA, these grid box dimensions were created. The active site of the CT domain was identified by examining the coordinates of the bound crystallographic inhibitor tepraloxydim. Binding affinity values (Δ*G*: kcal/mol) from the CT domain‐MMS and DNA‐MMS dockings served as control groups for comparative analysis with the docking results of CT domain‐HRM, CT domain‐HR, DNA‐HRM, and DNA‐HR. For the HRM, HR, and MMS dockings against both target receptors, the exhaustiveness parameter was set to 100, and the number of docking runs was set to 20. AutoDock Vina 1.2.5 was used to cluster post‐docking binding conformations of HRM and HR based on geometric similarity. These conformations were then ranked according to the binding affinity, with the conformation exhibiting the most favorable (most negative) binding free energy (Δ*G*: kcal/mol) being considered the top‐ranked docking pose. The top‐ranked docking poses of HRM and HR calculated for CT domain and DNA were qualitatively analyzed, rendered, and prepared on the Discovery Studio Visualizer (Biovia 2016).

### 2.6 Statistical Analysis

The statistical analysis of the results, which are shown as mean ± standard deviation, was conducted using SPSS version 23. For group comparisons and bivariate group correlations, one‐way ANOVA was used in conjunction with the Duncan test and Pearson correlation test, with significance levels of *p* ≤ 0.05 and *p* = 0.01, respectively.

## 3 Results and Discussion

### 3.1 Cyto‐Genotoxicity of HRM

**FIGURE 3:**
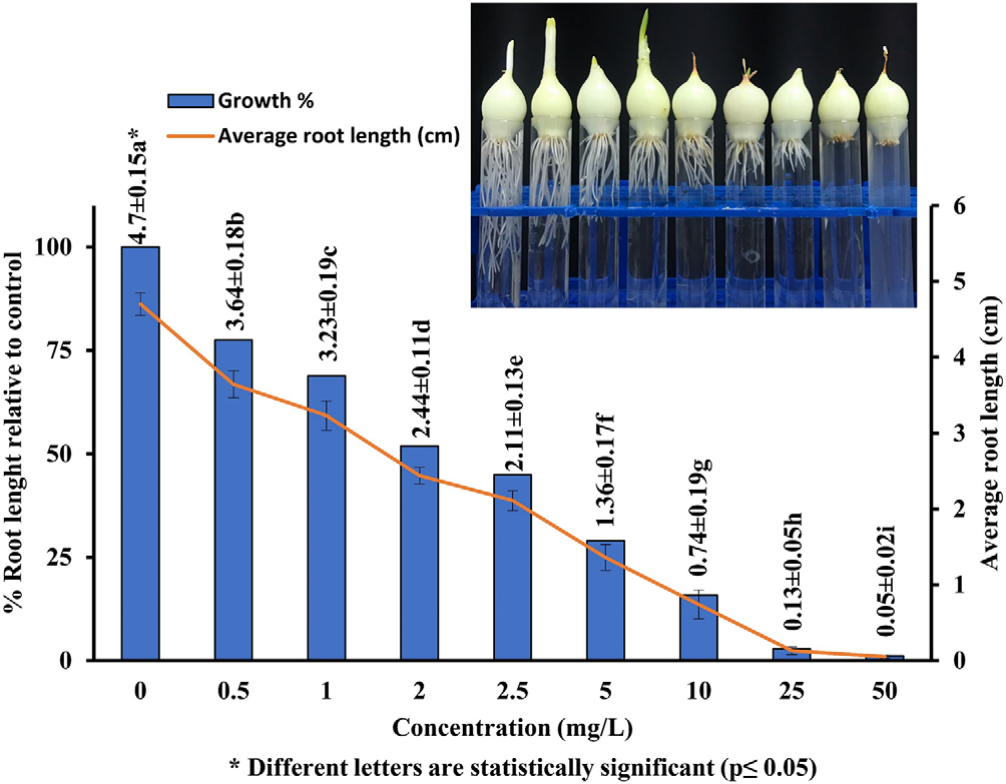
Root growth of *Allium cepa* roots in the control and groups exposed to HRM for 96 h.

The toxicity of HRM was assessed using the *A. cepa* root growth inhibition test (Figure 3). The EC_50_ concentration for HRM was calculated as 2.38 mg/L. The onion root lengths were significantly decreased in a dose‐dependent manner (*r* = −0.989) with increasing concentrations of HRM. Root growth inhibition was observed as follows: 0.5 mg/L (3.64 ± 0.18 cm, 22.55%), 1 mg/L (3.23 ± 0.19 cm, 31.28%), 2 mg/L (2.44 ± 0.11 cm, 48.09%), 2.5 mg/L (2.11 ± 0.3 cm, 55.51%), 5 mg/L (1.36 ± 0.17 cm, 71.06%), 10 mg/L (0.74 ± 0.19 cm, 84.26%), 25 mg/L (0.13 ± 0.05 cm, 97.23%), and 50 mg/L (0.05 ± 0.02 cm, 98.94%). The observed decrease in root length values due to HRM exposure can be attributed to the inhibition of meristematic activity at the apex (Scherer et al. 2019), interruption of the cell cycle (Fusconi et al. 2006), and inactivation of cell division enzymes (Silveira et al. 2017). Similar results after HRM exposure in plants were also reported in *Avena sativa* (Kim and Bendixen 1987), in *Zea mays* and *Glycine max* (Gronwald 1986), in *Sorghum bicolor* (L.) Moench (Vaughn 1987), in triticale, wheat, barley, rye, maize, oats, rice, couch grass, and two dicotyledonous species (Banas et al. 2011), and in *Alopecurus myosuroides* and *Lepidium sativum* (Buerge et al. 2015).

A significant decrease in the MI except at 1.19 mg/L in 72 h (Table 2) was observed with HRM exposure in a dose‐dependent (24 h *r* = −0.934, 48 h *r* = −0.954, 72 h *r* = −0.895, and 96 h *r* = −0.997) manner. A time‐dependent decrease in MI was also noted only for the 4.76 mg/L HRM concentration (*r* = −0.997). The decrease in MI at the highest dose of HRM was found to be even lower than that of the positive control (MMS). HRM statistically significantly increased the prophase index while decreasing the anaphase and telophase indices compared to the negative control group at the 72 and 96 h applications. The inhibition of the CHFR checkpoint, which delays or stops the cell from entering the metaphase, may be the cause of the rise in prophase cells (Magdaleno et al. 2015). The anaphase–telophase arrest may suggest that HRM is interfering with proteins that are essential for proceeding into the M‐phase (de Souza et al. 2023). A decrease in the MI in treatment groups after exposure to HRM has been reported previously on sorghum and oat (Kim and Bendixen 1987; Vaughn 1987). Similar results have also been reported after treating *A. cepa* root tip cells with other aryl phenoxy‐propionate herbicides such as clodinafop‐propargyl (Boumaza et al. 2016), diclofop‐methyl (Mesi and Kopliku 2013; Yüzbaşıoğlu et al. 2009), fluazifop‐P‐butyl (Ammar et al. 2022), and quizalofop‐p‐ethyl (Rosculete et al. 2018). The current study suggests that the decrease in MI induced by HRM may result from cell cycle inhibition, either by blocking DNA synthesis or preventing the cell cycle transition from G_1_ to S and from G_2_ to M (AA et al. 2000; Aslanturk 2024; Kim and Bendixen 1987; Sudhakar et al. 2001). Additionally, the reduction in mitotic activity or alterations in the duration of mitotic stages could be due to toxic chemical exposure (Hidalgo et al. 1989; Saxena et al. 2005) or anti‐mitotic effects, potentially through the inhibition of DNA polymerase and related cell cycle proteins (Hidalgo et al. 1989; Kaya et al. 2023; Turkoglu 2015). Therefore, it can be inferred that HRM reduces the MI of onion root cells due to its cytotoxic effects.

**TABLE 2:** Efficacy of HRM on *Allium cepa* roots' mitotic and phase indices.

Concentration (mg/L)	CCN	MI ± SD^a^	Phase index (%) ± SD^a^			
			Prophase	Metaphase	Anaphase	Telophase
Control‐24 h	5159	67.78 ± 1.15a	93.67 ± 1.22a	2.49 ± 0.68a	2.12 ± 0.44a	1.72 ± 0.28 ac
MMS‐10	5003	53.29 ± 1.16b	92.31 ± 0.92a	1.43 ± 0.27b	1.68 ± 0.45 ac	4.58 ± 0.7b
1.19	5193	65.67 ± 0.61c	96 ± 1.41b	1.44 ± 0.64b	1.32 ± 0.43bc	1.24 ± 0.47a
2.38	5158	64.78 ± 1.37c	93.78 ± 1.54a	2.3 ± 0.56 ac	1.79 ± 0.73 ac	2.12 ± 0.42c
4.76	5244	43.33 ± 1.84d	97 ± 0.6b	1.72 ± 0.4bc	0.75 ± 0.16b	0.53 ± 0.21d
Control‐48 h	5226	69.67 ± 1.18a	94.57 ± 1.24a	1.64 ± 0.44ab	1.59 ± 0.42ab	2.2 ± 0.53a
MMS‐10	5053	51.69 ± 1.51b	92.78 ± 1.42b	1.35 ± 0.34ab	1.29 ± 0.7a	4.59 ± 0.69b
1.19	5322	65.42 ± 1.33c	94.28 ± 1.05ab	2.05 ± 0.61b	1.69 ± 0.28ab	1.98 ± 0.72a
2.38	5212	58.38 ± 1.24d	93.95 ± 1.24ab	1.81 ± 0.39a	2.23 ± 0.65b	2 ± 0.49a
4.76	5276	35.62 ± 0.72e	99.89 ± 0.11c	0.06 ± 0.1c	0.06 ± 0.1c	—
Control‐72 h	5190	68.28 ± 1.11a	96.75 ± 0.72a	1.19 ± 0.26a	0.93 ± 0.37a	1.13 ± 0.26a
MMS‐10	5005	45.31 ± 1.73b	90.13 ± 2.79b	2.46 ± 1.02b	3.13 ± 0.79b	4.28 ± 1.24b
1.19	5208	68.22 ± 3.58a	99.45 ± 0.3c	0.29 ± 0.2c	0.15 ± 0.1c	0.11 ± 0.01c
2.38	5301	65.08 ± 1.93c	99.19 ± 0.45c	0.44 ± 0.26 ac	0.2 ± 0.13c	0.17 ± 0.18c
4.76	5269	32.88 ± 1.2d	99.01 ± 0.56c	0.76 ± 0.17 ac	0.11 ± 0.06c	0.11 ± 0.05c
Control‐96 h	5256	67.59 ± 0.96a	94.67 ± 0.5a	1.52 ± 0.34a	1.62 ± 0.51a	2.19 ± 0.16a
MMS‐10	5220	44.31 ± 1.69b	92.39 ± 1.3b	1.74 ± 0.46a	1.79 ± 0.65a	4.08 ± 0.7b
1.19	5311	63.92 ± 1.37c	96.94 ± 0.74c	0.86 ± 0.25b	1.03 ± 0.25b	1.17 ± 0.25c
2.38	5269	42.66 ± 1.4b	98.61 ± 0.72d	0.59 ± 0.53b	0.45 ± 0.23c	0.36 ± 0.19d
4.76	5190	23.23 ± 1.09d	98.49 ± 0.45d	0.92 ± 0.37b	0.26 ± 0.23c	0.34 ± 0.19d

Abbreviations: CCN, counted cell numbers; MI, mitotic index; SD, standard deviation.

^a^ Distinct letters within identical columns for every treatment interval exhibit statistical significance (*p* ≤ 0.05).

**FIGURE 4:**
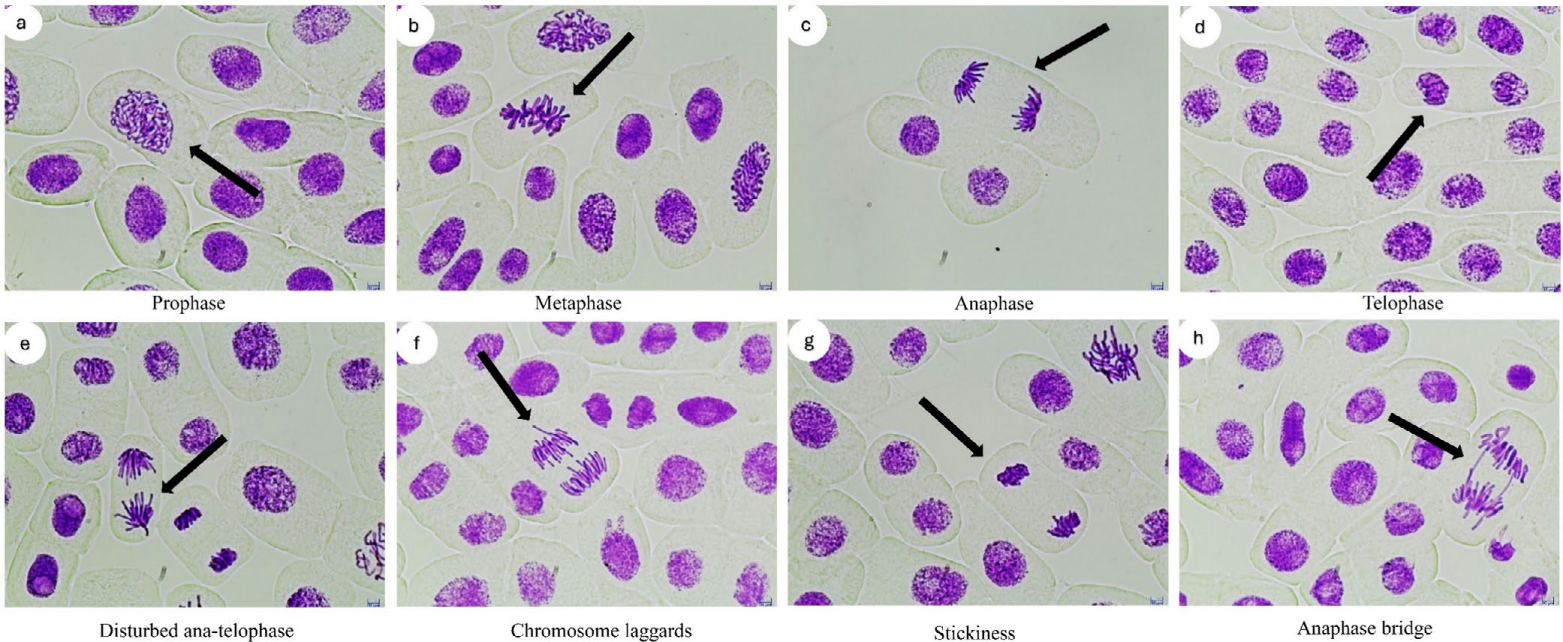
Mitotic stages and chromosomal aberrations in *Allium cepa* roots exposed to HRM. Scale bars = 10 μm with 400× magnification.

HRM statistically significantly increased the CAs (except at 4.76 mg/L in 48 h due to extensive toxicity) compared to the negative control group at 24 and 48 h treatments by using the Allium ana–telophase test (Table 3). The highest CAs were found at 11.2% at 2.38 mg/L in 24 h, while the lowest ones were found at 4.4% at 1.19 mg/L in 24 h. Ana–telophase chromosome anomalies (stickiness, chromosome laggards, anaphase bridges, and disturbed ana–telophase, Figure 4) induced by HRM could not be observed in 72 and 96 h treatments due to the decrease in anaphase and telophase indices compared to the negative control. The most frequently observed type of CAs induced by HRM was stickiness (4.6% at 2.38 mg/L in 24 h). Sticky chromosomes (Figure 4g) are a type of chromatid aberration that can result from depolymerization, condensation, or fragmentation of DNA or chromosome breakage and irregular chromosome coiling (Barman and Ray 2023; Fatma et al. 2018), potentially leading to the formation of chromosomal bridges (Haq et al. 2016). Disturbed ana–telophase (Figure 4e) and chromosomal laggards (Figure 4f) may result from irregular chromosomal movements due to the disruption of spindle formation, which prevents motor proteins on the spindle fibers from transferring the corresponding chromosomes to the poles (Das and Ray 2024; Kizilkaya et al. 2023). Herbicides' clastogenic effects may cause chromosomal breakage or intermingling, unequal chromatid exchange, dicentric chromosomes, or differences in replication enzymes, which can result in the formation of anaphase bridges (Figure 4h) (AA et al. 2000; Beatriz Andrioli et al. 2022; Das et al. 2021). *A. cepa* root tip cells treated with other aryl phenoxy‐propionate herbicides such as Topik (containing 80 g/L clodinafop‐propargyl; Boumaza et al. 2016), Illoxan (containing 284 g/L diclofop‐methyl; Yüzbaşıoğlu et al. 2009), Fusilade Max (containing Fluazifop‐P‐butyl 12.5% EC; Ammar et al. 2022), and quizalofop‐P‐ethyl (Ranjan et al. 2018; Sharma and Vig 2012; Yildiz and Arikan 2008) were shown to induce CAs along with different concentrations and treatment time intervals.

**TABLE 3:** HRM‐induced chromosomal anomalies in the root meristematic cells of *Allium cepa* .

Concentration (ppm)	Ana–telophase anomalies %					
	CCN	DAT	CL	S	AB	TA ± SD^a^
Control‐24 h	500	0.6	0.4	0.4	0.8	2.2 ± 0.45a^a^
MMS‐10	500	1.8	5.4	8.8	3.2	19.2 ± 0.84b
1.19	500	0.6	0.8	1.6	1.4	4.4 ± 0.55c
2.38	500	2.6	2.2	4.6	1.8	11.2 ± 0.84d
4.76	500	1	1.4	1.6	0.6	4.6 ± 0.89c
Control‐48 h	500	1.4	0.6	1	0.8	3.8 ± 0.45a
MMS‐10	500	1.6	4.8	8.8	3.4	18.6 ± 0.89b
1.19	500	1.2	1.8	1.4	0.8	5.2 ± 0.84c
2.38	500	1.4	1.6	1.2	1.4	5.6 ± 0.55c
4.76	500	—	—	—	—	—

Abbreviations: AB, anaphase bridge; CCN, counted cell number; CL, chromosome laggards; DAT, disturbed anaphase–telophase; S, stickiness; SD, standard deviation; TA, total anomalies.

^a^ Distinct letters within identical columns for every treatment interval exhibit statistical significance (*p* ≤ 0.05).

**FIGURE 5:**
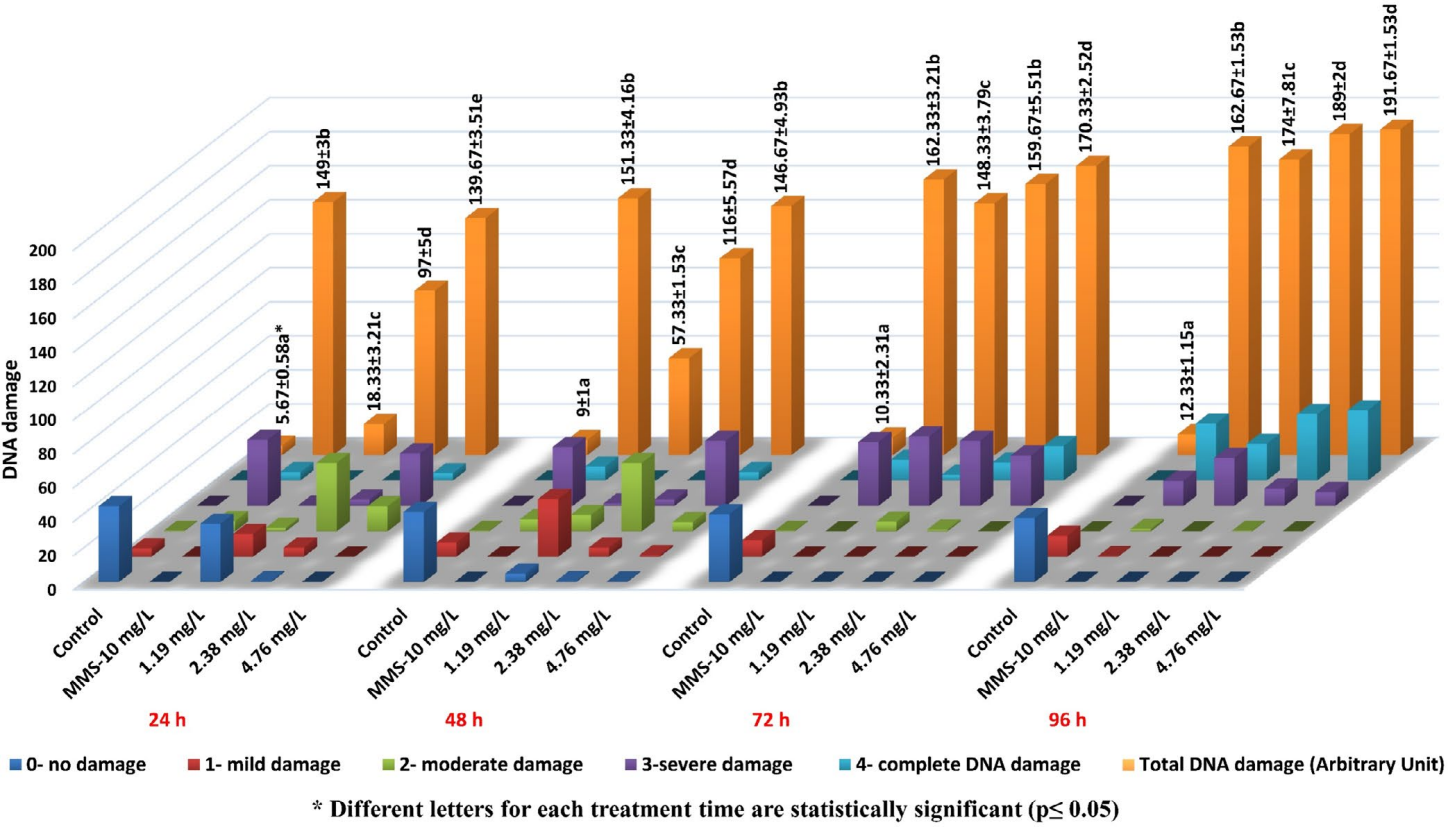
HRM‐induced DNA damage in *Allium cepa* roots.

HRM statistically significantly increased the DNA damage in *A. cepa* root tips compared to the negative control group at all treatment periods in the comet assay (Figure 5). The DNA damage increased upon exposure to HRM was found both concentration‐ (*r* = 0.984 for 24 h, *r* = 0.979 for 48 h, *r* = 0.936 for 72 h, and *r* = 0.830 for 96 h) and time‐dependent (*r* = 0.976 for 1.19 mg/L, *r* = 0.984 for 2.38 mg/L, and *r* = 0.970 for 4.76 mg/L). Although no studies have investigated the effect of HRM on DNA damage using the comet assay in the plant test material, there are studies that HRM induces oxidative stress by promoting ROS production, generating free radicals, and causing enzyme functional alterations in non‐target organisms such as in fresh turnip and black radish (Koksal et al. 2018), in cereal leaves (Lukatkin et al. 2013), in rat testis (Olayinka and Ore 2015), in mudflat crab (Xu et al. 2023), and in zebrafish embryos (Park et al. 2020) and larvae (Liu et al. 2018).

### 3.2 HRM‐CT Domain Interactions

**FIGURE 6:**
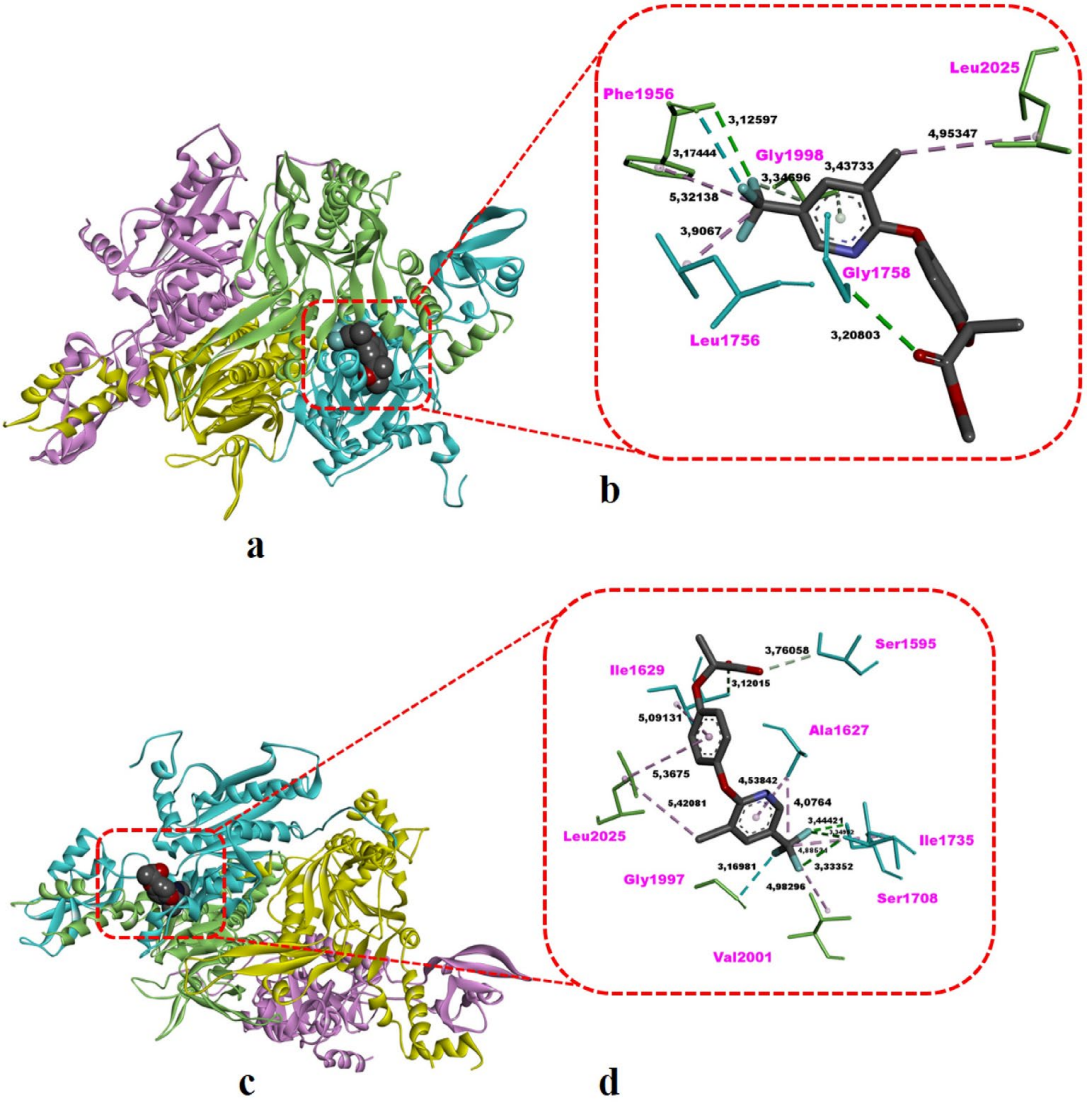
Top‐ranked post‐docking conformation of HRM pro‐herbicide complexed with the CT domain of yeast ACC enzyme (a, b). (a) 3D overview of the CT domain–HRM complex; (b) Detailed interaction view of HRM within the active site of the CT domain. Green dashed lines indicate hydrogen bonds, light purple dashed lines indicate hydrophobic contacts, and light cyan dashed lines indicate halogen interactions. Top‐ranked post‐docking conformation of HR in complex with the CT domain of the yeast ACC enzyme (c, d). (c) 3D overview of the CT domain–HR complex; (d) Detailed interaction view of HR within the active site of the CT domain. Green dashed lines denote hydrogen bonds, honeydew dashed lines denote carbon–hydrogen bonds, light purple dashed lines denote hydrophobic contacts, and light cyan dashed lines denote halogen interactions. In both images (b, d), non‐bonded interaction distances (Å) are shown in bold black. Images were created using DS Studio v16 software.

The intermolecular interactions and binding affinity of HRM, the pro‐herbicide, with the CT domain of the yeast ACC enzyme are detailed in Table 4 and illustrated in Figure 6a,b. Amino acid residue interactions of HRM in the catalytic pocket (dimeric interface) of the CT domain revealed two H‐bonds with Gly1758 and Phe1956, two carbon–hydrogen (non‐classical) bonds with Gly1998, three hydrophobic contacts with Leu1756, Phe1956, and Leu2025, and a halogen (fluorine) interaction with Phe1956 (Table 4 and Figure 6b). HRM demonstrated a favorable binding interaction (Δ*G* = −7.40 kcal/mol) with the CT domain, comparable to the strong binding affinity of tepraloxydim (Δ*G* = −7.83 kcal/mol), the crystal inhibitor, as shown in Table 4.

**TABLE 4:** Docking scores (kcal/mol) and CT domain interactions of HRM, HR, and the reference ligand tepraloxydim at the dimeric interface of yeast ACC.

						Hydrophobic interaction	
Compound	Molecular weight (g/mol)	Receptor	Δ*G*_best_ (kcal/mol)	Classical H‐bond	Non‐classical H‐bond (carbon–hydrogen)	Alkyl/π‐alkyl interaction	Halogen (fluorine)
Tepraloxydim (crystal inhibitor)	341.83	CT domain^a^	−7.83	Ile1735 (3.27 Å), Gly1998 (2.88 Å)	Gly1997 (3.14 Å)	Ala1627 (3.46 Å), Leu1705 (4.55 Å), Ala1712 (3.93 Å), Tyr1738 (4. 22 Å) Val1975 (5.36 Å), Val2001 (4.12 Å, 5.39 Å), Val2002 (4.74 Å), Val2024 (4.15 Å), Leu2025 (4.97 Å)	—
HRM	375.72	CT domain	−7.40	Gly1758 (3.20 Å), Phe1956 (3.12 Å)	Gly1998 (3.34 Å, 3.44 Å)	Leu1756 (3.90 Å), Phe1956 (5.32 Å), Leu2025 (4.95 Å)	Phe1956 (3.17 Å)
HR	340.08	CT domain	−7.62	Ile1629 (3.12 Å), Ser1708 (3.44 Å), Ile1735 (3. 33 Å, 3. 34 Å)	Ser1595 (3.76 Å)	Ala1627 (4.07 Å, 4.53 Å), Ile1629 (5.09 Å), Ile1735 (4. 88 Å), Leu2025 (5.36 Å, 5.42 Å)	Gly1997 (3.16 Å), Val2001 (4.98 Å)

^a^ (PDB ID: 3K8X): Structure of the CT domain of ACC complexed with tepraloxydim. Δ*G*_best_: Binding free energy (kcal/mol) of the most favorable pose. The underlined amino acid residues (Ile1735, Ala1627, Gly1997, Gly1998, Val2001, and Leu2025) indicate that HRM and HR could bind to the same residues as the crystal inhibitor, tepraloxydim, in docking simulations, demonstrating the effectiveness of the docking search algorithm.

### 3.3 HR–CT Domain Interactions

The analysis of interactions of HR in the catalytic site of the CT domain (Table 4 and Figure 6c,d, respectively) showed that HR formed four H‐bonds with Ile1629, Ser1708, and Ile1735, one carbon–hydrogen bond with Ser1595, six hydrophobic contacts with Ala1627, Ile1629, Ile1735, and Leu2025, and two halogen (fluorine) interactions with Gly1997 and Val2001 (Table 4 and Figure 6d). When compared with the crystal inhibitor tepraloxydim, HR, the active herbicide, exhibited a highly strong binding ability against the CT domain (Δ*G* = −7.62 kcal/mol), which is comparable with that of tepraloxydim (Δ*G* = −7.83 kcal/mol) (Table 4). The molecular mechanism underlying the stronger interaction of HR, in comparison to the pro‐herbicide HRM, involves the formation of a greater number of H‐bonds (with Ile 1629 and Ile1735) as well as halogen interactions (with Gly1997 and Val2001) (Table 4 and Figure 6d), highlighting its stronger binding ability to the CT domain of the ACC enzyme.

### 3.4 HRM–DNA Interactions

**FIGURE 7:**
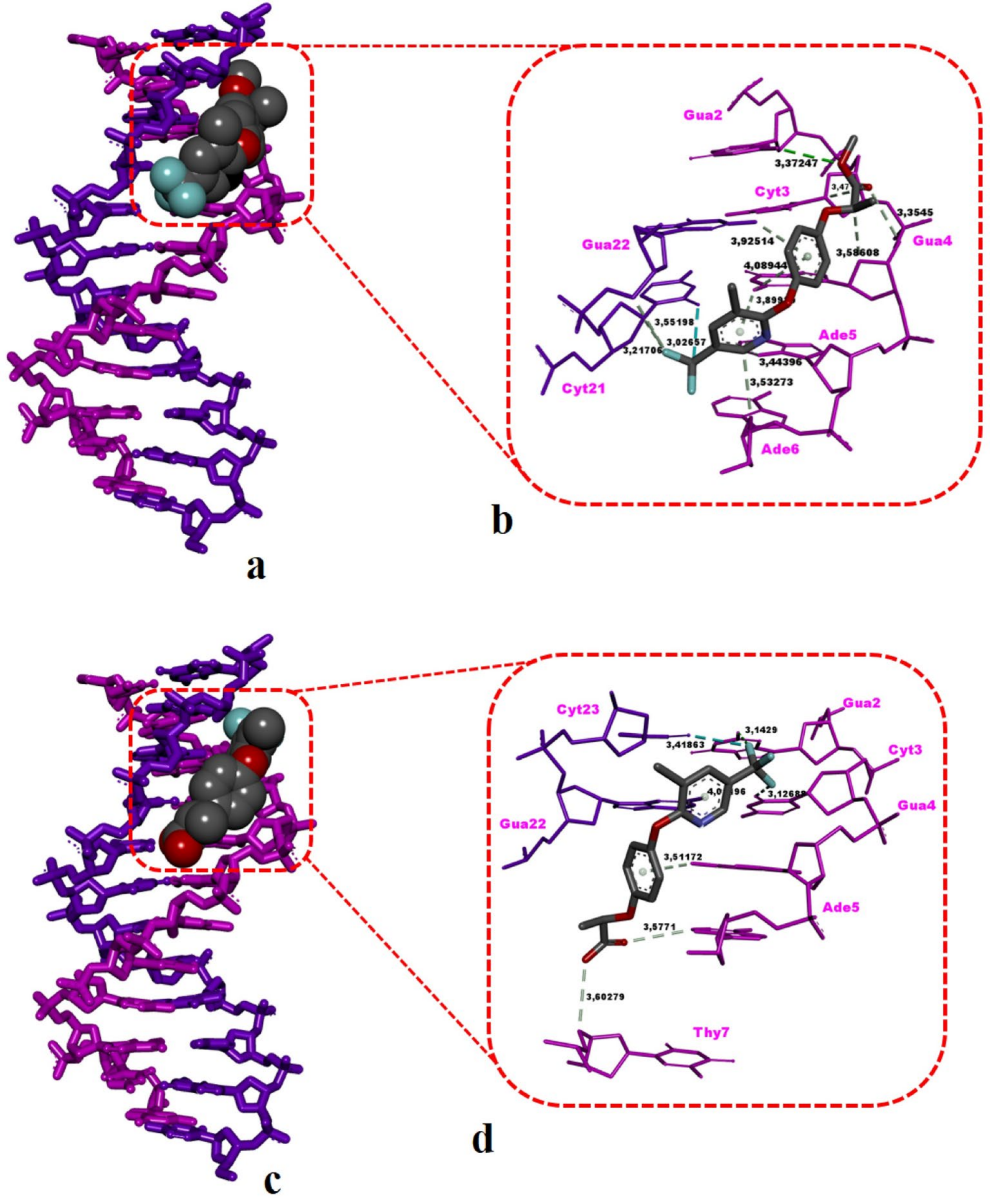
Binding mode of HRM with the double‐stranded DNA fragment (a, b). (a) 3D image of HRM's minor groove recognition mode (DNA receptor in stick mode and HRM in CPK mode). Note that HRM fits snugly into the minor groove of the DNA molecule. (b) Zoomed‐in interaction diagram of HRM with DNA nucleotides. Green and light green dashed lines denote classical and non‐classical hydrogen bonds, respectively, while aqua dashed lines indicate halogen bonds. Binding mode of haloxyfop‐R (HR) active herbicide with the double‐stranded DNA fragment (c, d). (c) 3D image of HR's minor groove recognition mode (DNA receptor in stick mode and HR in CPK mode). Note that HR fits snugly into the minor groove of the DNA molecule. (d) Zoomed‐in interaction diagram of HR with the DNA nucleotides. Green and light green dashed lines denote classical and non‐classical hydrogen bonds, respectively, while aqua dashed lines indicate halogen bonds. Non‐bonded interaction distances (Å) are shown in black next to each dashed line. Figures were created using DS Studio Visualizer v16.

DNA nucleotides interact with HRM, along with the DNA‐binding mode and types of HRM–DNA non‐bonded interactions, as detailed in Table 5 and illustrated in Figure 7a,b. HRM exhibited its optimal binding conformation in the minor groove of DNA, involving nucleotides Gua2, Cyt3, Gua4, Ade5, Ade6, Cyt21, and Gua22. HRM snugly fit into the minor groove, stabilized by one classical hydrogen bond (Gua2), 10 carbon‐hydrogen bonds (Cyt3, Gua4, Ade5, Ade6, Cyt21, and Gua22), and a halogen (fluorine) interaction (Table 5 and Figure 7b). The binding free energy of HRM against the dsDNA was highly favorable (Δ*G* = −7.26 kcal/mol) compared to the experimental positive control MMS (Δ*G* = −3.03 kcal/mol) (Table 5). Of note, the simultaneous binding of HRM with both strands of DNA and its minor groove recognition mode, similar to the DNA‐reactive mutagen MMS, reflect the genotoxic nature of this agent (Figure 7b). Furthermore, it has been determined that the interaction frequency of HRM with DNA nucleotides follows the pattern G=C>A, indicating a higher affinity toward GC‐rich regions.

**TABLE 5:** Binding free energies (kcal/mol), binding modes, and DNA nucleotide interaction details, including nonbonded distances (Å), for methyl MMS, HRM, and HR, as determined by docking experiments.

							Miscellaneous
Compound	Receptor	Δ*G*_best_ (kcal/mol)	Binding mode	Classical H‐bond	Non‐classical H‐bond	Halogen (fluorine)	Pi‐sulfur
MMS (positive control)	B‐DNA	−3.03	Minor groove	—	Ade6 (3.65 Å, 3.33 Å), Cyt21 (3.29 Å), Gua22 (3.39 Å)	—	Ade5 (5.79 Å), Cyt21 (5.94 Å)
HRM	B‐DNA	−7.26	Minor groove	Gua2 (3.37 Å)	Cyt3 (3.47 Å), Ade5 (3.44 Å), Ade6 (3.53 Å), Gua4 (3.58 Å, 3.35 Å, 4.08 Å, 3.89 Å), Cyt21 (3.21 Å), Gua22 (3.92 Å, 3.55 Å)	Cyt21 (3.02 Å)	—
HR	B‐DNA	−7.34	Minor groove	Gua2 (3.14 Å)	Ade5 (3.57 Å), Thy7 (3.60 Å), Gua4 (3.51 Å), Gua22 (4.90 Å)	Cyt3 (3.12 Å), Cyt23 (3.41 Å)	—

Abbreviations: MMS, methyl methanesulfonate—experimental positive control; Δ*G*_best_, Binding free energy (kcal/mol) of the most favorable docking pose.

### 3.5 HR–DNA Interactions

The post‐docking DNA nucleotide interactions of HR, its DNA‐binding mode, and the HR–DNA non‐bonded interactions are shown in Table 5 and Figure 7c,d. The DNA recognition mode of HR was determined as minor groove binding, which is mediated by nucleotides Gua2, Cyt3, Gua4, Ade5, Thy7, Gua22, and Cyt23. The snug fit of HR against the minor groove was stabilized by a H‐bond (Gua2), four carbon–hydrogen (non‐classical) bonds (Ade5, Thy7, Gua4, Gua22), and two halogen (fluorine) interactions (Table 5 and Figure 7d). The binding score of HR with the dsDNA molecule was observed to be highly favorable (Δ*G* = −7.34 kcal/mol), showing a significantly stronger binding ability compared to the experimental mutagen MMS (Δ*G* = −3.03 kcal/mol) (Table 5). In a similar manner with HRM, genotoxic HR is also capable of interacting simultaneously with the complementary strands of DNA and exhibits a minor groove recognition mode specific to the known mutagen MMS (Figure 7b,d). It can be concluded that the interaction frequency of HR with DNA nucleotides is in the order of G=C>A=T, suggesting its specificity as a binding agent for GC‐rich regions.

According to the docking results, both HRM and HR exhibited energetically favorable binding to the active site (dimeric interface) of the CT domain of the yeast ACC enzyme involved in fatty acid metabolism (Table 4 and Figure 6). Additionally, the binding strength of both molecular forms of the herbicide to the CT domain is closely comparable to the crystal inhibitor, tepraloxydim (Table 4). However, the binding strength of HR to the CT domain is slightly higher than that of HRM, and this has been attributed to the formation of a greater number of H‐bonds and halogen (fluorine) interactions (Table 4). Therefore, it can be inferred that the active herbicide form HR strongly inhibits the ACC enzyme in *A. cepa* meristematic root cells, thereby impeding fatty acid biosynthesis and consequently inhibiting cell proliferation by disrupting the synthesis of new cell membranes. This is consistent with the significantly reduced MI observed throughout all treatment durations and at high concentrations (2.38 and 4.36 ppm) in *A. cepa* root cells, along with an extreme increase in the number of cells unable to progress beyond prophase (Table 1). Furthermore, the blockade of fatty acid synthesis due to ACC inhibition can negatively impact cellular energy metabolism, thereby slowing down or completely inhibiting mitotic division (De Carvalho and Caramujo 2018).

Based on dsDNA interaction results, it has been determined that the dsDNA binding modes of both HRM and HR involved minor groove recognition (Figure 7). The affinity of HRM against dsDNA was Δ*G* = −7.26 kcal/mol, while that of the active herbicide form HR was slightly stronger with a Δ*G* = −7.34 kcal/mol (Table 5). It has been determined that both herbicide forms exhibit specificity toward GC‐rich regions in dsDNA and could bind simultaneously with the complementary strands of the biomolecule (Figure 7b,d). There is no existing study in the literature regarding the adverse interactions of HRM or HR with the DNA molecule; therefore, our study represents a novel contribution both experimentally and computationally. The stable complexes formed by HRM and its rapidly hydrolyzed form HR with the DNA molecule (Table 5 and Figure 7b,d) may lead to the inhibition of the processivity of the helicase enzyme (responsible for separating both strands of DNA) in these regions during replication. The progression of the replication fork may be impeded in such ligand–receptor hybrid regions where the continuity of replication is disrupted, leading to the emergence of DNA breaks and, consequently, the formation of DNA fragments. The extreme numbers of total chromosome aberrations observed at the median concentration (2.38 ppm) after 24 h treatment in *A. cepa* root meristematic cells (Table 3), along with the significant DNA fragmentation observed in the comet assay across all treatment durations and concentrations (Figure 5), may arise from the DNA‐damaging stable complexes formed between HRM, HR, and DNA (Table 5). In summary, considering computational docking and experimental observations, the active herbicide HR may show its cytotoxic and genotoxic effects in *A. cepa root* cells in a bimodal manner through significant ACC blockade as well as stable binding with DNA. Nevertheless, the residual amounts of pro‐herbicide form (HRM) that remain inactive within the organism may also be responsible to a certain extent for the cytotoxic and genotoxic effects observed in *A. cepa* meristematic cells.

## 4 Conclusion

The findings of this study confirmed that HRM had cyto‐genotoxic effects on *A. cepa* root tip cells by causing an increase in DNA damage and CAs while reducing root development and MI. In molecular docking experiments, it has been determined that the intracellularly active herbicide, HR, forms a highly tight binding complex with the CT domain of yeast ACC. Moreover, it was observed that HR exhibited energetically favorable binding to GC‐rich regions in the dsDNA molecule. These findings suggest that the underlying mechanism of the cyto‐genotoxic effects of HRM observed in *A. cepa* root cells may involve not only the inhibition of the crucial role played by the ACC enzyme in fatty acid biosynthesis but also the ability of HR, the active herbicide, to form a stable complex with the DNA molecule. Thus, the HR enantiomer may act as a multimodal cytotoxic agent in *A. cepa* meristematic cells. Nevertheless, further research is required to completely comprehend HRM's cyto‐genotoxic processes.

## Author Contributions

**Recep Liman:** conceptualization, data curation, formal analysis, investigation, methodology, validation, writing – review and editing, writing – original draft, supervision. **Emine Kılıç:** formal analysis, methodology. **Erman Salih İstifli:** conceptualization, methodology, validation, writing – review and editing. **Yudum Yeltekin Uğur:** formal analysis, methodology. **İbrahim Hakkı Ciğerci:** conceptualization, investigation, methodology, resources, writing – review and editing.

## Conflicts of Interest

The authors declare no conflicts of interest.

## Data Availability

The data that support the findings of this study are available from the corresponding author upon reasonable request.
